# Loss of *Inpp5d* has disease‐relevant and sex‐specific effects on glial transcriptomes

**DOI:** 10.1002/alz.13901

**Published:** 2024-06-26

**Authors:** Luke C. Dabin, Holly Kersey, Byungwook Kim, Dominic J. Acri, Daniel Sharify, Audrey Lee‐Gosselin, Cristian A. Lasagna‐Reeves, Adrian L. Oblak, Bruce T. Lamb, Jungsu Kim

**Affiliations:** ^1^ Department of Medical & Molecular Genetics Indiana University School of Medicine Indianapolis Indiana USA; ^2^ Stark Neuroscience Research Institute Indiana University School of Medicine Indianapolis Indiana USA; ^3^ Medical Neuroscience Graduate Program Indiana University School of Medicine Indianapolis Indiana USA; ^4^ Department of Anatomy Cell Biology & Physiology Indiana University School of Medicine Indianapolis Indiana USA; ^5^ Center for Computational Biology and Bioinformatics Indiana University School of Medicine Indianapolis Indiana USA; ^6^ Department of Radiology and Imaging Sciences Indiana University School of Medicine Indianapolis Indiana USA

**Keywords:** Alzheimer, dementia, *Inpp5d*, microglia, network analysis, neurodegeneration, sex difference, single‐cell, transcriptomics

## Abstract

**INTRODUCTION:**

*Inpp5d* is genetically associated with Alzheimer's disease risk. Loss of *Inpp5d* alters amyloid pathology in models of amyloidosis. *Inpp5d* is expressed predominantly in microglia but its function in brain is poorly understood.

**METHODS:**

We performed single‐cell RNA sequencing to study the effect of *Inpp5d* loss on wild‐type mouse brain transcriptomes.

**RESULTS:**

Loss of *Inpp5d* has sex‐specific effects on the brain transcriptome. Affected genes are enriched for multiple neurodegeneration terms. Network analyses reveal a gene co‐expression module centered around *Inpp5d* in female mice. *Inpp5d* loss alters Pleotrophin (PTN), Prosaposin (PSAP), and Vascular Endothelial Growth Factor A (VEGFA) signaling probability between cell types.

**DISCUSSION:**

Our data suggest that the normal function of *Inpp5d* is entangled with mechanisms involved in neurodegeneration. We report the effect of *Inpp5d* loss without pathology and show that this has dramatic effects on gene expression. Our study provides a critical reference for researchers of neurodegeneration, allowing separation of disease‐specific changes mediated by *Inpp5d* in disease from baseline effects of *Inpp5d* loss.

**Highlights:**

- Loss of *Inpp5d* has different effects in male and female mice.
- Genes dysregulated by *Inpp5d* loss relate to neurodegeneration.
- Total loss of *Inpp5d* in female mice collapses a conserved gene co‐expression module.
- Loss of microglial *Inpp5d* affects the transcriptome of other cell types.

## 1 INTRODUCTION

Alzheimer's disease (AD) is characterized by neuropathology, including aggregation and deposition of misfolded amyloid beta (Aβ) and tau proteins. To better understand the genetic risk for sporadic AD, genome‐wide association studies (GWAS) have identified protective and risk variants across the human genome, opening new routes for investigating the etiology of AD. For example, a meta‐analysis of two AD GWAS (*n* = 54,162) revealed an intronic single‐nucleotide polymorphism (SNP; rs35349669) in *INPP5D* that is associated with AD risk.^1^ Subsequently, a second intronic SNP (rs61068452‐G) was found to be associated with increased cerebral blood flow in the left angular gyrus,^2^ a site where blood flow is impaired in AD.^3^, ^4^

*INPP5D* encodes an SH2 domain–containing inositol phosphatase known as SHIP‐1.^5^ This enzyme hydrolyzes phosphatidylinositol trisphosphate (PIP3) to phosphatidylinositol bisphosphate (PIP2), inhibiting activation of the AKT serine/threonine kinase (AKT) pathway and negatively affecting cell growth and proliferation. *INPP5D* is expressed primarily in myeloid cells, and its ablation in mice leads to the proliferation of peripheral immune cells that subsequently invade vital organs, leading to hyperplasia, atrophy, and premature death.^6^ SHIP‐1 also contains two NXPY motifs, which, on phosphorylation, can be recognized by Src homology 2 (SH2) domain–containing proteins or other phosphotyrosine‐binding proteins, such as Src Homology and Collagen proteins (SHC).^7^ The C‐terminus contains a proline‐rich sequence recognized by Src homology 3 (SH3) domain–containing proteins, notably Growth Factor Receptor Bound Protein 2 (GRB2).^8^ SHC and GRB2 are upstream of Ras activation, and their sequestration by SHIP‐1 has been proposed to further inhibit cell proliferation through the Mitogen Activated Protein Kinase (MAPK) pathway.^9^ During development, *Inpp5d* regulates the differentiation trajectories of chondrocytes, epidermal cells, macrophages, hematopoietic cells, and T cells.^10^, ^11^, ^12^, ^13^, ^14^ In summary, SHIP‐1 is an important regulator of peripheral immune cell proliferation and functions through both its phosphatase activity and as a molecular scaffold in signaling complexes.^15^

In the brain, *Inpp5d* is thought to be expressed mainly in microglia, which serve as the brain's resident immune cells and are currently thought to be major players in predisposing against, responding to, and regulating AD pathology.^16^ Recent research has revealed that *Inpp5d* was upregulated in amyloid plaque–associated microglia in 5XFAD models of familial AD and that ablation of *Inpp5d* in these mice reduced amyloid pathology.^17^, ^18^ Conversely, another study found that amyloid pathology was increased when *Inpp5d* was inducibly depleted in PSAPP mice.^19^ Recently, haploinsufficiency of *Inpp5d* was shown to rescue microglial function in NLGF mice lacking both copies of *Tyrobp*.^20^ Although these studies suggest conflicting effects of *Inpp5d* on neuropathology in amyloid mouse models, little is known about the normal function of *Inpp5d* in microglia under physiological conditions without amyloid pathology and how different cell types in the brain react to the loss of *Inpp5d* expression.

Although previous work has focused on reducing the expression of *Inpp5d* in mouse models of amyloid, we aimed to determine the effects of both partial and total *Inpp5d* loss on the brain transcriptome at single‐cell resolution in both male and female wild‐type mice in the absence of amyloid. This study design with wild‐type mice is a critical step in understanding the function of *Inpp5d* without the confounding effect of amyloid pathology on cell transcriptomes, as is the case in humans before AD pathology starts.

## 2 METHODS

### 2.1 Animals

All experiments were approved by the Institutional Animal Care and Use Committee at Indiana University. Mice were housed and maintained in a 12‐h/12‐h light/dark cycle with food (Purina Lab Diet, 5K52) and water ad libitum. C57BL/6J (B6; The Jackson Laboratory, 000664) and B6.129S6(C)‐*Inpp5d* ^tm1.1Wgk^/J (*Inpp5d* ^+/−^; mice with 50% reduced *Inpp5d* expression; The Jackson Laboratory, 028269) were used to breed heterozygous and homozygous knockout mice as well as wildtype littermates. As homozygous knockout mice are not viable after 7–12 weeks (https://www.jax.org/strain/028269), per consultation with our veterinary staff we set experimental timepoints at 6 weeks.

**RESEARCH IN CONTEXT**

1. Systematic review: The authors reviewed the literature on single‐cell transcriptomics data to understand the role of *Inpp5d* in brains through PubMed search. Although the research on the role of *Inpp5d* in the context of Alzheimer's disease is gaining momentum, there has been no robust dataset for its physiological function.
2. Interpretation: This study reveals cell‐type–specific transcriptomic alterations occurring upon the loss of *Inpp5d*. These effects occur at the level of gene networks, particularly those that regulate actin remodeling, and are amplified in females. The loss of a microglial *Inpp5d* gene has unexpected effects on gene expression in other cell types, notably astrocytes.
3. Future directions: Our data inform future studies aiming to investigate the role of *Inpp5d* in brain under physiological conditions, which will be critical to distinguish it from its role under pathological conditions. The interplay between microglia and astrocytes in *Inpp5d*‐deficient brains emerges for further investigation.

### 2.2 Single‐cell RNA library preparation

Two male and two female 6‐week‐old mice per genotype (*Inpp5d* ^+/+^, *Inpp5d* ^+/−^, *Inpp5d* ^−/−^) were anesthetized with 1.2% 2,2,2‐tribromoethanol (Avertin) and transcardially perfused with ice‐cold 0.1 M phosphate‐buffered saline (PBS). The brain was extracted and the cerebellum and olfactory bulb were removed from the right hemisphere, which was immediately finely minced on ice and transferred to a polypropylene tube containing 10 mL ice‐cold Accutase (Gibco, A11105‐01). Tissue was incubated with rocking at 4°C for 30 min and then pelleted through centrifugation at 300 *g* for 5 min at 4°C. Supernatant was aspirated and samples were resuspended in 5 mL ice‐cold wash buffer (1X Hanks' Balanced Salt Solution (HBSS) (Gibco, 14175095), 0.04% Bovine Serium Albumin (BSA) (Miltenyi, 130‐091‐376) and gently dissociated through pipetting the suspension up and down 15 times using a 5 mL serological pipette. After all samples were processed, the cloudy upper suspension was transferred to a fresh 50 mL polypropylene tube and the remaining clumps of tissue were further dissociated using an additional 5 mL ice‐cold wash buffer. The cloudy upper suspension was again transferred to the 50 mL polypropylene tube, and the remaining tissue was dissociated using an additional 2 mL wash buffer and a 1000 µL pipette. Cell strainers—70 and 40 µm (Corning, 352350 and 352340)—were moistened with 5 mL ice‐cold wash buffer, and samples were strained through into fresh polypropylene tubes; each filter was rinsed with an additional 5 mL of ice‐cold wash buffer. Cell suspensions were pelleted through centrifugation at 300 *g* for 10 min at 4°C. The supernatant was aspirated and cells were resuspended in 1080 µL of degassed MACS Myelin Removal Buffer (1X DPS (Gibco 14190250), 0.5% BSA (Miltenyi, 130‐091‐376)) and transferred to 2 mL DNA LoBind tubes (Eppendorf, 022431048). Anti‐myelin beads (120 µL) Miltenyi, 130‐096‐733) were added to each sample and mixed thoroughly by pipetting. Samples and beads were incubated for 15 min at 4°C with rotating. Samples were transferred to 15 mL polypropylene tubes and washed with 10 mL myelin‐removal buffer, and then pelleted through centrifugation at 300 *g* for 10 min at 4°C. Supernatant was aspirated and samples were resuspended in 2 mL myelin‐removal buffer.

LS columns (Miltenyi, 130‐042‐401) were loaded into a QuadroMACS separator on a MACS MultiStand with Tube Rack (Miltenyi, 130‐090‐976 and 130‐042‐303 and 130‐091‐052). Columns were equilibrated with three successive 1 mL volumes of myelin‐removal buffer, which were collected in 15 mL polypropylene tubes. Cell suspensions were loaded onto columns, and myelin‐depleted cell suspensions were washed through using four successive 1 mL volumes of myelin‐removal buffer. Effluent was made up to 10 mL total volume and cells were pelleted through centrifugation at 300 *g* for 5 min at 4°C. Cells were then pelleted through centrifugation at 300 *g* for 5 min at 4°C before resuspension in 200 µL resuspension buffer (1X Dulbecco's Phosphate‐Buffered Saline (DPS) (Gibco 14190250); 0.04% BSA (Miltenyi, 130‐091‐376)), another centrifugation step using the same settings, and finally resuspension in 50 µL resuspension buffer. An aliquot of cells was stained with trypan blue and the cell concentration and viability were determined by manual counting using a disposable hemocytometer slide (C‐Chip, SKC, Inc., Covington, GA) and the 40X objective of an EVOS XL Core microscope. Cell viability was >94%. Single‐cell suspensions were mixed with a single‐cell master mix containing lysis buffer and reverse transcription reagents following the Chromium Next GEM Single Cell 3′ Reagent Kits User v3.1 (Dual Index) Guide, CG000315 Rev A (10X Genomics, Pleasanton, CA). Each single‐cell mix was then loaded into a single well of a Single Cell Chip G and run on the Chromium Controller for GEM generation and barcoding. Sample processing and library preparation were performed according to the manufacturer's instructions using the Chromium Next GEM Single Cell 3′ v3.1 dual index kit (10X Genomics, Inc.) and SPRIselect paramagnetic bead‐based chemistry (Beckman Coulter Life Sciences, Indianapolis, IN). The cDNA and library quality were assessed using the Agilent 2100 Bioanalyzer and a High Sensitivity DNA kit (Agilent Technologies, Santa Clara, CA), and the final library concentration was determined using a Qubit Fluorometer and the dsDNA HS assay kit (Thermo Fisher Scientific, Waltham, MA). Sequencing was carried out on a NovaSeq 6000 v1.5 S2 100‐cycle chip (Illumina) with 28‐10‐10‐91 read setup.

### 2.3 Single‐cell RNA‐sequencing data analysis

Processing of the sequencing data was performed with the *CellRanger* pipeline (v4.0.0, 10X Genomics). First CellRanger used bcl2fastq (https://support.illumina.com/) to demultiplex raw base sequence calls generated from the sequencer into sample‐specific FASTQ files. Feature Barcode analysis function in CellRanger then quantified each feature in each cell: briefly, the FASTQ files were aligned to an appropriate reference genome with RNAseq aligner STAR. The aligned reads were traced back to individual cells and the expression level of individual features was quantified based on the number of unique molecular indices (UMIs) detected in each cell. The filtered feature‐cell barcode matrices (including the hashtag count matrix) generated by CellRanger were loaded into SoupX^21^ (v1.5.1) in Rstudio (v1.4.1717) running R (v4.0.4) and cleaned using default settings. Heterotypic doublets were removed using DoubletFinder^22^ (v2.0.3). Cleaned data were then loaded into Seurat^23^ (v4.0.5).

During quality control, cells with >10% mitochondrial reads, or unique reads below 200 or above median + 1.5 times SD (3900), were excluded from analysis. Data were normalized using SCTransform (v0.3.3) and cells were clustered using the first 26 principal components based on an Elbow Plot. Cluster marker genes were identified using the FindConservedMarkers function. We used the scMCA function (v0.2.0) provided by the Mouse Cell Atlas^24^ to annotate cluster identity. Differences in cluster occupancy between genotypes and sex were tested using a two‐way Analysis of Variance (ANOVA) with Šídák's multiple comparisons test in GraphPad Prism (v9.1.0). Data from female and male samples were analyzed separately in the same way, with 22 and 25 principal components used for clustering each respective data set.

Differential gene expression analysis was performed using a Wilcoxon ranked‐sum test per cluster, with Bonferonni adjustment of *p*‐values. High‐dimensional weighted gene correlation network analysis (hdWGCNA) was performed using hdWGCNA^25^, ^26^, ^27^ (v0.2.04). CellChat^28^ (v1.1.2) was used to infer intercluster signaling using default package settings. Both hdWGCNA and CellChat were performed on astrocyte, endothelial cell, microglia, and oligodendrocyte clusters after merging all clusters of these types.

**FIGURE 1:**
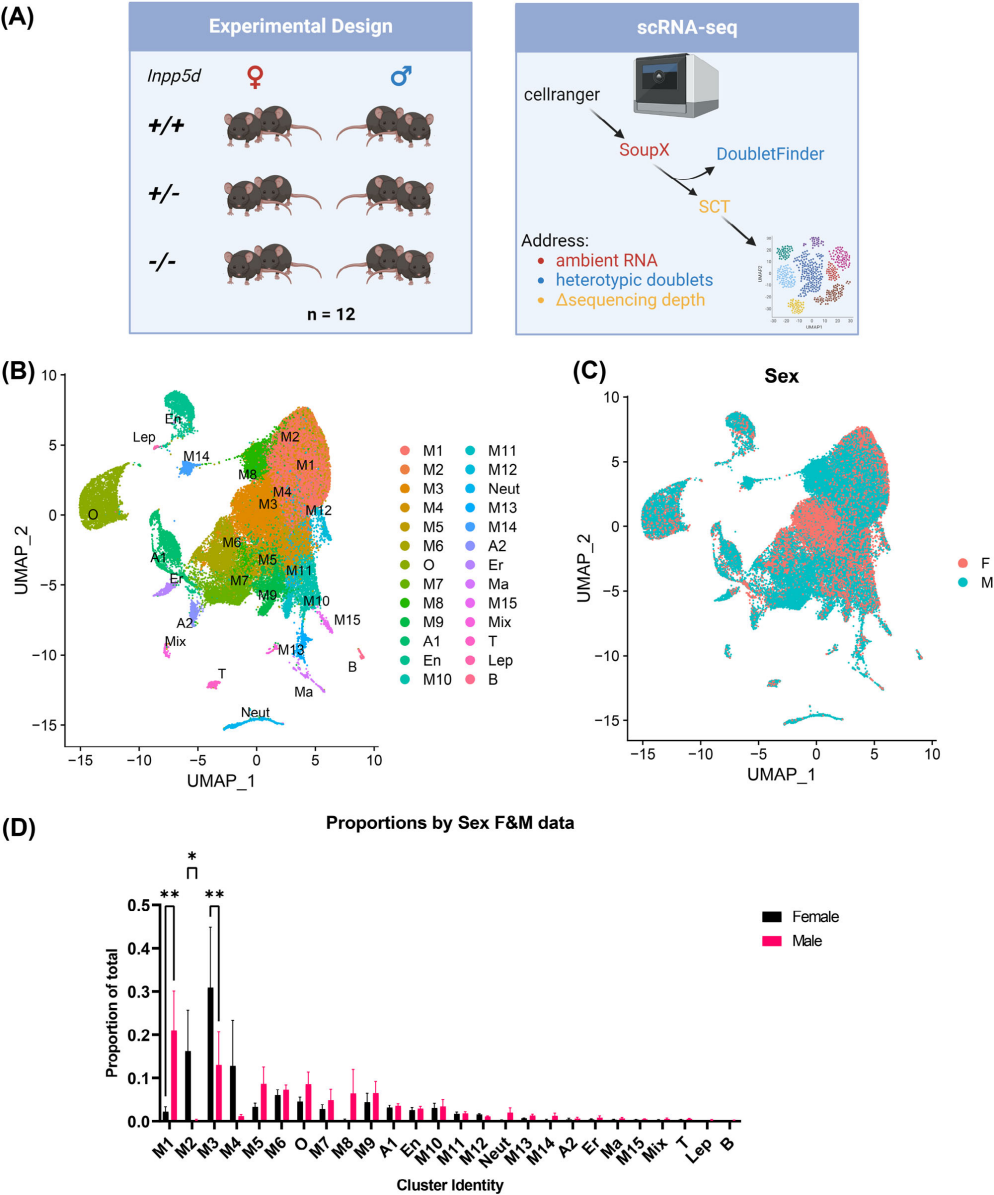
Cluster proportions in single‐cell RNAseq data from *Inpp5d* knockout mouse brain differ between sexes. (A) Experimental design: two mice per sex per genotype (*n* = 12) were used to generate single‐cell RNAseq data. These were processed using CellRanger, before removal of ambient RNA using SoupX, removal of heterotypic doublets using DoubletFinder, and adjustment for differences in sequencing depth per sample using SCTransform. (B) Annotated Uniform Manifold Approximation and Projection (UMAP) plot of 57,742 cells after quality control including microglia (M1‐M15), oligodendrocyte (O), astrocyte (A1‐A2), endothelial (En), neutrophil (Neut), erythrocyte (Er), macrophage (Ma), mixed (Mix), T (T), leptomeningeal (Lep), and B (B) cell clusters. (C) UMAP showing the sample origin per cell based on sex. (D) Proportions of cells in each cluster based on sex. Analysis of Variance (ANOVA) using Šídák's multiple comparisons test was used to determine nominally significant changes (*P < 0.05, **P < 0.01, error bars show Standard Error of the Mean (SEM)).

Figures 1A and 5A were created using https://BioRender.com.

**FIGURE 2:**
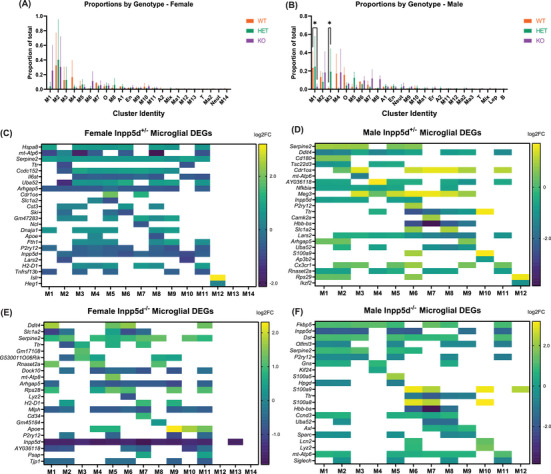
Clustering of *Inpp5d* scRNAseq data separated by sex reveals differences in cell populations due to genotype. (A) Proportions of cells in each cluster in female data. (B) Proportions of cells in each cluster in male data. ANOVA using Šídák's multiple comparisons test was used to determine significant nominally significant changes (*p < 0.05, **p < 0.01, error bars show SEM). (C–F) Representative heatmaps of microglial DEGs in female and male *Inpp5d^+/−^ and Inpp5d^−/−^* mice compared to wild‐type littermates. Color shade represents log2‐fold‐change, all shaded tiles represent Bonferroni‐significant DEGs. Blank tiles did not pass an adjusted significance threshold of 0.05.

Raw and cellranger‐processed data have been deposited in the NIH GEO database, scRNA‐seq data (GSE254999).

## 3 RESULTS

### 3.1 Loss of *Inpp5d* has sex‐ and genotype‐specific effects on microglial populations

To ensure that we captured sufficient biological variation in our study, we bred male and female *Inpp5d* ^+/+^, *Inpp5d* ^+/−^, and *Inpp5d* ^−/−^ mice and aged them to 6 weeks (two per sex per genotype, total *n* = 12). Given that *Inpp5d* is predominantly expressed in microglia, we used a custom neuron‐depleting single‐cell dissociation protocol^29^ to isolate cells from the mouse brain. After correction for ambient RNA contamination, doublet inference and removal, and quality control (see Methods), 57,742 cells were taken forward for analysis (Figure 1A). First, we performed clustering on the data and identified 26 unique clusters (Figure 1B, Table S1). After annotation using the single‐cell Mouse Cell Atlas (scMCA), we classified these cells as microglia (M1‐15), oligodendrocytes (O), astrocytes (A1‐2), neutrophils (Neut), erythrocytes (Er), endothelial cells (En), macrophages (Ma), leptomeningeal cells (Lep), T cells (T), B cells (B), and a small population of ambiguous “mixed” cells (Mix). We confirmed the selective expression of *Inpp5d* in microglial clusters and the small number of myeloid cells and erythrocytes present in our study (Figure S1), as well as the total loss of expression in *Inpp5d* ^−/−^ mice. Before performing any advanced downstream analyses, we first tested whether there were different numbers of cells in each cluster due to genotype. Homozygous loss of *Inpp5d* caused a statistically significant increase in the number of cells in microglial cluster 4 (*p* = 0.039; Figure S2), whereas other clusters demonstrated differences in the number of cells per genotype; these differences did not pass the significance threshold after adjustment for multiple comparisons. Strikingly, when we plotted the proportion of cells in each cluster by sex, we detected significant differences in the proportions of M1 (*p* = 4.4 × 10^−3^), M2 (*p* = 0.0348) and M 3 (*p* = 8.4 × 10^−3^) microglia (Figure 1C,D). We thus decided to split the data into male‐ and female‐derived data sets to control for sex as a confounding biological variable in downstream analyses.

### 3.2 Loss of *Inpp5d* causes extensive dysregulation of genes linked to multiple forms of neurodegeneration

We reclustered and annotated the 26,593 cells in the female‐only data set, resolving 23 clusters. These included microglia (M1‐14), oligodendrocytes (O), astrocytes (A1‐2), neutrophils (Neut), endothelial cells (En), macrophages, T cells (T), and a small population of “mixed” cells (Mix) (Table S2 and Figure S3). The 31,149 cells in the male‐only data set formed 25 clusters composed of microglia (M1‐12), oligodendrocytes (O), astrocytes (A1‐2), neutrophils (Neut), endothelial cells (En), erythrocytes (Er), leptomeningeal cells (Lep), macrophages (Ma1‐3), T‐ cells (T), B cells (B), and a small population of “mixed” cells (Mix) (Table S3, Figure S4, and Figure S5). We observed no significant differences in the number of cells per cluster per genotype in the female data set (Figure 2A). Of interest, compared to wild‐type, there was a significant decrease in cell proportions from male *Inpp5d^−/−^* mice in microglial cluster 1 (*p* = 0.0129), and a significant increase in cell proportions from male *Inpp5d^+/−^* mice in microglial cluster 3 (*p* = 0.0323) (Figure 2A).

**FIGURE 3:**
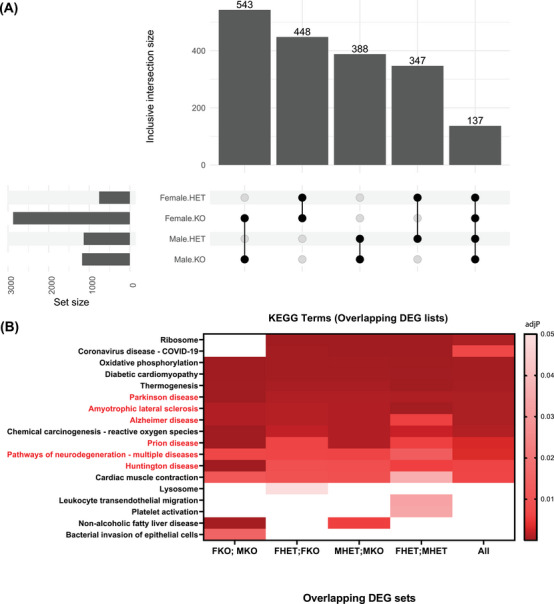
Differential gene expression signatures based on genotype and sex all show ontological enrichment with neurodegenerative diseases. (A) Upset plot showing the number of unique differentially expressed genes (set size) across all clusters within each group compared to wild‐type and the size of the overlap between comparisons of interest. Differential expression was determined by Wilcoxon Rank Sum test with Bonferroni adjustment for multiple comparisons (HET, heterozygous knockout; KO, homozygous knockout). (B) Heatmap of Gene Ontology (GO) enrichment analysis with Kyoto Encyclopedia of Genes and Genomes (KEGG) terms reveals that all five overlapping lists of DEGs are enriched for genes in KEGG lists for Parkinson disease, amyotrophic lateral sclerosis, Alzheimer's disease, prion disease, pathways of neurodegeneration, and Huntington disease (*p*‐value adjustment performed using the Benjamini‐Hochberg method; all significantly enriched terms are shown per comparison—none were excluded).

We then performed differential gene expression analysis for each cluster in both female and male datasets between *Inpp5d* ^+/+^ & *Inpp5d* ^+/−^, and *Inpp5d* ^+/+^ & *Inpp5d* ^−/−^ mice (Table S4), as well as enrichment analysis of the differentially expressed genes (DEGs) for each cluster using Gene Ontology (GO) and Kyoto Encyclopedia of Genes and Genomes (KEGG) terms (Table S5). The direction of differential expression varied between each cluster, sex, and *Inpp5d* genotype. Across both sexes, in astrocytes, 347 genes were upregulated, whereas 607 genes were downregulated; in endothelial cells, 179 genes were upregulated and 859 genes were downregulated; in microglia, 2502 genes were upregulated and 3816 genes were downregulated; and in oligodendrocytes, 281 genes were upregulated and 1021 genes were downregulated. Representative heat maps of microglial DEGs are shown in Figure 2C‐F.

We were interested in identifying which DEGs were (1) unique to *Inpp5d* depletion in females or males only, (2) unique to *Inpp5d^+/−^* or *Inpp5d^−/−^* mice regardless of sex, and (3) DEGs shared between all comparisons. There were almost three times more DEGs (2874 DEGs) in female *Inpp5d* ^−/−^ mice than in any other comparison group, whereas there were overlaps in DEGs between male and female *Inpp5d* ^−/−^ mice (543 DEGs), *Inpp5d* ^+/−^ and *Inpp5d* ^−/−^ DEGs in female mice (448 DEGs), and *Inpp5d* ^+/−^ and *Inpp5d* ^−/−^ DEGs in male mice (388 DEGs). A total of 137 DEGs overlapped between heterozygous and homozygous knockout mice of both sexes (Figure 3A, Figure S6, and Table S6).

**FIGURE 4:**
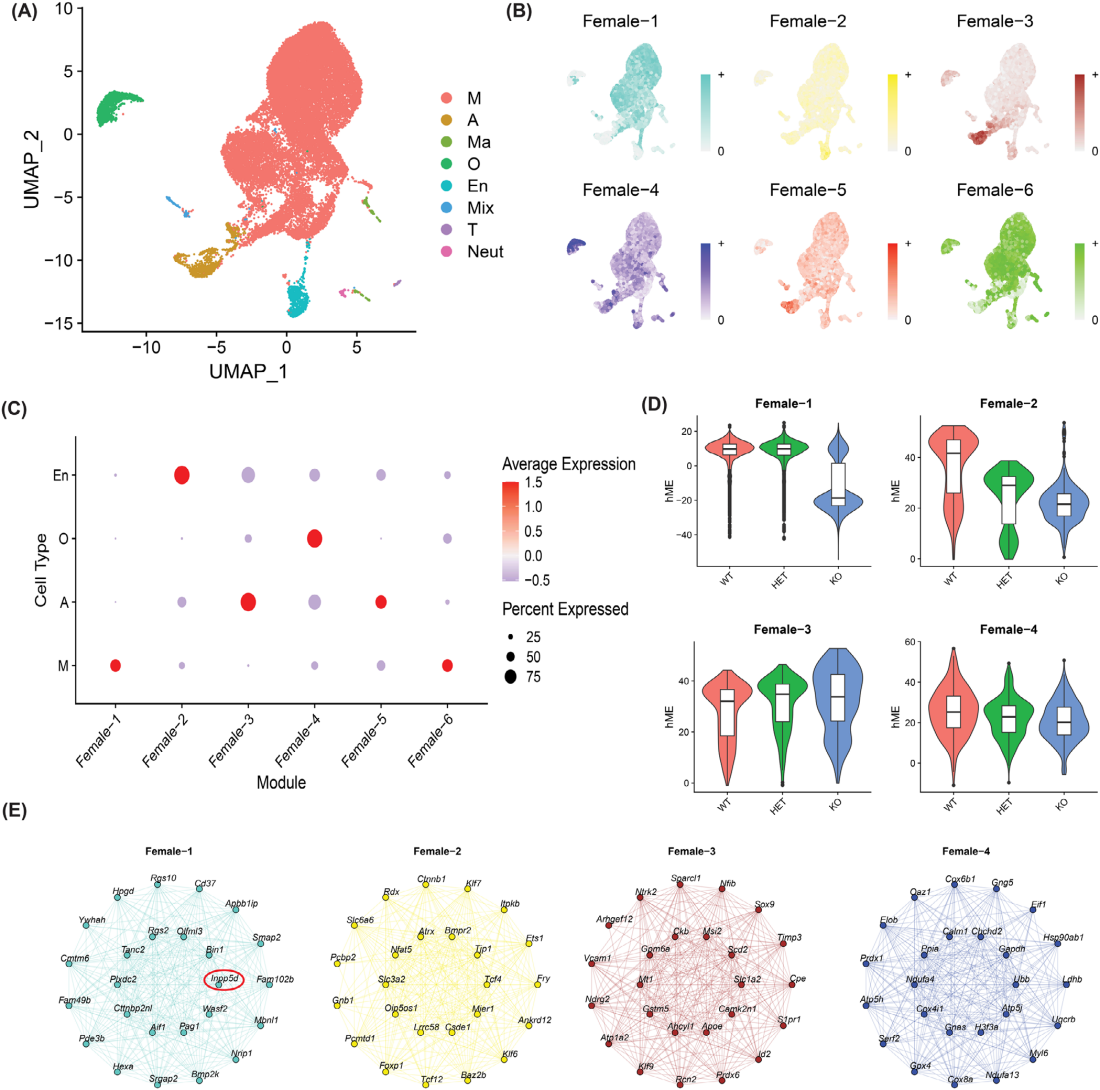
High‐dimensional WGCNA reveals cell‐type specific co‐expression gene modules in female mice, including a microglial module with *Inpp5d* at its center. (A) UMAP showing seven clusters of merged cell types. The four largest clusters—microglia (M), astrocytes (A), oligodendrocytes (O), and endothelial cells (En)—were used in this analysis. (B) Feature plot of expression of Hub genes (top 10% of genes as ranked using Maximal Clique Centrality) per module (Female 1–6). (C) Dotplot showing scaled expression of each module across cell types. Average scaled expression level scales with blue (low) to red (high) shading. Size of dots represents the percent of cells in each cell type expressing the module. (D) Eigenvalues of the four modules where module eigengene changes with genotype in their assigned cell types. (E) Network plots of the 25 most central genes in each module. In Female‐1, *Inpp5d* is the most central gene and is circled in red. Nodes are labeled with the genes they represent; edges represent gene–gene co‐expression.

To clarify the effects of such extensive dysregulation of gene expression, we performed enrichment analyses on each of these overlapping gene sets. Strikingly, despite these mice being on a wild‐type (C57BL/6J) background and not being crossed with any animal model of neurodegeneration, ontology terms for multiple neurodegenerative diseases (Parkinson's disease, amyotrophic lateral sclerosis, Alzheimer's disease, prion disease, pathways of neurodegeneration, and Huntington's disease) were significantly enriched within DEG lists across all comparisons (Figure 3B; Figure S7 and Table S7).

### 3.3 Loss of *Inpp5d* has dramatic effects on gene regulatory networks in female mice compared to male mice

**FIGURE 5 alz13901-fig-0005:**
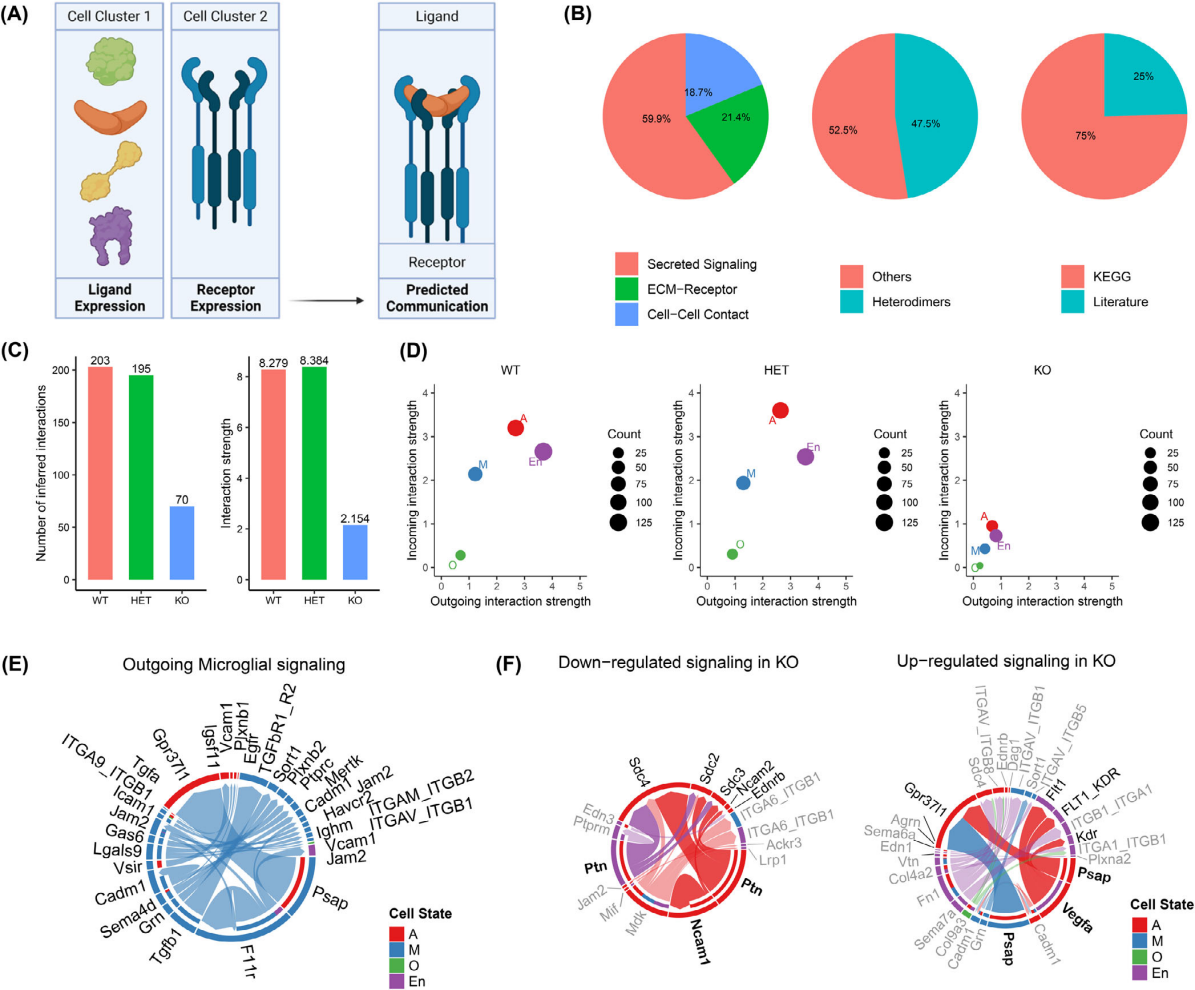
Total loss of *Inpp5d* significantly reduces global signaling between cell types in female mice but increases proastrocytic signaling between microglia and astrocytes. (A) CellChat uses the expression of 2021 ligands and receptors between pairs of cells in scRNAseq data sets to infer cell–cell communication. (B) Characteristics of ligand–receptor interactions in the CellChat database. (C) The number and strength of inferred interactions drops substantially on homozygous loss of *Inpp5d* in female mice. (D) Numbers and strengths of incoming and outgoing interactions decrease in all cell types on total loss of *Inpp5d* in female mice. (E) Outgoing microglial signaling pathways in all female mice. Most signals occur between microglial and astrocytes or other microglia. (F) Chord plots showing pathways with decreased and increased signaling in *Inpp5d* ^−/−^ mice compared to wild‐type. Plot circumference shows outgoing signaling from cell type (outer band) to recipient cell type (inner band). Width of outer bands represents numbers of cells per pathway. All pathways shown are significantly dysregulated; some bands are emphasized for the purpose of visualization.

Having cataloged the effects of *Inpp5d* depletion on individual genes across multiple cell types, we performed high‐dimensional weighted gene correlation network analysis (hdWGCNA) to determine whether modules of coexpressed genes were affected by the loss of *Inpp5d*. To assess such broad changes, we merged clusters based on cell type (Figure 4A) and ran hdWGCNA, focusing only on microglia, astrocytes, oligodendrocytes, and endothelial cells. Other cell types were not included in this analysis because they were sparsely represented. We identified six modules of coexpressed genes in the female data set (Figure 4B; Table S8) and tested the expression of these gene modules across cell types. There was striking enrichment of each module in a particular cell type; for example, the Female‐1 module was highly expressed in microglia (Figure 4C). We then[Fig alz13901-fig-0005] plotted the eigenvalue of each module within its cell type between genotypes. The eigenvalues of the Female‐1, Female‐2, and Female‐4 modules were dramatically reduced in *Inpp5d* ^−/−^ microglia, endothelial cells, and oligodendrocytes, respectively (Figure 4D). The eigenvalue of the Female‐2 module also decreased in *Inpp5d* ^+/−^ endothelial cells, whereas the eigenvalue of the Female‐3 module increased only in *Inpp5d^+/−^* astrocytes. We selected the Female‐1 module for further analysis because its expression is selectively enriched in microglia (Figure 4C), and its eigenvalue decreases upon the loss of *Inpp5d* (Figure 4D). We ranked the 5056 genes in this module by centrality and found that the gene with the highest intramodule connectivity was indeed *Inpp5d*, suggesting that Female‐1 is composed of genes coexpressed with or regulated by *Inpp5d* (Figure 5D; Table S8).

We then performed the same experiments with the male data set, merging clusters into their shared cell types (Figure S8). We identified five co‐expressed modules (Figure S8 and Table S9), including a Male‐1 module that was also expressed mostly in microglia. Unlike in females, *Inpp5d* was much less central in this module (256/5798), and there was no notable change in the eigenvalue according to genotype.

### 3.4 Loss of *Inpp5d* in microglia may alter signaling from microglia to other cell types

We found that the loss of a microglial‐expressed gene can cause changes in gene module preservation in other cell types (Figure 5D). These findings prompted us to evaluate changes in cell–cell communication in our data using CellChat.^28^ CellChat can predict the probability of 2021 validated molecular interactions between receptors and ligands across groups of cells (Figure 5A–B). To assess broad changes across cell types, we again performed an analysis after merging the clusters belonging to the same cell type. With respect to the female data set, we observed a decrease in the total number of interactions between cell types with loss of *Inpp5d* from wild‐type (203 interactions) to heterozygous knockout (195 interactions) to homozygous knockout (70 interactions) (Figure 5C, Table S10). The sum probability of interactions (interaction strength) also decreased from wild‐type (8.279) and heterozygous knockout (8.384) to homozygous knockout (2.154) (Figure 5C). Of interest, both the incoming and outgoing interaction strengths decreased dramatically in *Inpp5d^−/−^* astrocytes, microglia, and endothelial cells, suggesting that the loss of Inpp5d in microglia affects signaling in other cell types (Figure 5D). Given that *Inpp5d* is expressed exclusively in microglia (Figure S1), we decided to infer outgoing signaling from microglia to other cell types. It is notable that microglia signal mostly to other microglia and astrocytes (Figure 5E).

Because the greatest decrease in incoming and outgoing signaling occurred in female *Inpp5d^−/−^* mice, we visualized the significantly up‐ and downregulated interactions between female *Inpp5d* wild‐type and knockout mice. We found that inferred pleiotrophin (PTN) and neural cell adhesion molecule 1 (NCAM1) signaling from astrocytes to astrocytes was decreased (Figure 5F). Conversely, prosaposin (PSAP) signaling from microglia to astrocytes, and PSAP and vascular endothelial growth factor A (VEGFA) signaling from astrocytes to astrocytes/endothelial cells, respectively, were increased (Figure 5F).

We also applied this analysis to the male data set and initially observed the opposite of what we observed in the female data set—a general increase in interaction strength in *Inpp5d* ^−/−^ mice, with the greatest increase occurring in astrocytes and endothelial cells (Figure S9). The number of interactions between cell types in the wild‐type (246 interactions) and heterozygous knockout (207 interactions) increased in the homozygous knockout mice (359 interactions). Similarly, the total sum probability of interactions in the wild‐type (6.331) and heterozygous knockout (6.482) increased to 10.628 in the homozygous knockout mice. When we focused on differences between genotypes, we observed significant down‐ and upregulation of multiple pathways (Table S11). However, again, we observed decreases in PTN signaling from astrocytes and endothelial cells in *Inpp5d* ^−/−^ mice and increases in PSAP signaling to astrocytes and VEGFA signaling from astrocytes to endothelial cells. We also inferred decreased junctional adhesion molecule A (F11R) signaling between microglia and an increase in NCAM1 signaling between astrocytes. Taken together, these findings indicate that decreases in PTN and increases in PSAP and VEGFA signaling may occur in both female and male *Inpp5d* ^−/−^ mice, whereas there is an opposite direction of NCAM1 communication probability between the astrocytes of each sex. Changes in the activity of these pathways based on sex and genotype could explain the changes we observed in the non‐microglial cell types we discussed.

## 4 DISCUSSION

In this article, we reported novel reference single‐cell transcriptome data sets for *Inpp5d^+/−^* and *Inpp5d* ^−/−^ mice using a protocol that maximized the capture of microglia. Given the increasing evidence that microglia play a critical role in AD pathogenesis^30^ and that *Inpp5d* is exclusively expressed in microglia, we believe that our data will be invaluable resources for the research community for designing future experiments. This is particularly timely, as interest in the role of *INPP5D*/SHIP1 in AD as a therapeutic target is only increasing.^31^, ^32^ No study has modeled the effect of total loss of *Inpp5d*, as this leads to death before sufficient AD‐like pathology has developed in mouse models. Our study is thus unique as we can study the effects of total deletion of *Inpp5d* on the brain transcriptome. By not crossing *Inpp5d* ^−/−^ mice with any models of amyloidosis, we were able to investigate the effect of *Inpp5d* haploinsufficiency on the brain transcriptome in the absence of pathology—in other words, modeling the effects prior to disease onset. Therefore, these data represent a baseline effect of *Inpp5d* loss, which other researchers can use to refine their findings and prune out any changes resulting from the loss of *Inpp5d* under normal conditions.

We found a striking difference in gene expression patterns between male and female mice upon the deletion of *Inpp5d*. Our data suggest that any transcriptomic study of *Inpp5d* manipulation should ensure that both sexes are represented with sufficient statistical power. We also identified an *Inpp5d*‐centric gene co‐expression module in female microglia containing 5056 genes, which may contain novel candidates for future functional studies. Finally, we inferred altered gene expression in non‐microglial cells, and changes in communication between cell types in both male and female *Inpp5d* ^−/−^ mice. Although we confirmed that *Inpp5d* is expressed only in microglia and other immune cells in the brain, its deletion seems to affect the transcriptome of non‐microglial cells. We conclude that non‐microglial cell types may also be worthy of further research when studying *Inpp5d* in models of neurodegeneration.

Of interest, we found significant differences in cluster cell proportions between males and females. There is some precedent for sex having effects on microglial morphology, gene expression, and function.^33^, ^34^, ^35^ Although there are ways of integrating data to remove the effects of such variables, we decided against this analysis approach to avoid the loss of findings linked to sex as an important biological variable. When the data were segregated by sex, we observed significant changes in cell proportions in microglial clusters 1 and 3 based on genotype. Cells in M1 were greatly decreased in *Inpp5d* ^−/−^ mice and seem to be redistributed, albeit not significantly, across M7 and M8. As the largest cluster in healthy young mice, M1 may represent homeostatic microglia. Two top marker genes for M1 are homeostatic markers *Cx3cr1* and *P2ry12*, whereas M7 shows high expression of activation marker *Tyrobp*.^36^, ^37^, ^38^ M8 shows high expression of *Lpl*, a gene recently implicated in myelin‐scavenging activated microlia.^39^ This suggests that *Inpp5d* loss drives a shift away from a homeostatic state. However, as our sample size was reduced to *n* = 2 per genotype and sex, and as variance across proportions in clusters M1 and M3 are high, we are hesitant to draw any firm conclusions from these distributions. Instead, we clustered the data and analyzed differential gene expression separately for each sex and focused on the overlaps between comparisons. Among the 137 genes dysregulated in all comparisons, several have been linked to microglial activation (*Hsp90ab1, Fth1, Sall1, Irf8, Cts3, Fus, Hexb, Apoe, Tyrobp, Lyz2*) or actin remodeling (*Actb, Macf1, Tns1, Ssh2, Tmsb4x, Srgap2, Rcsd1*), which are both processes that can be mediated by *Inpp5d*.^6^, ^20^ It seems likely that cytoskeletal remodeling would mediate chemotaxis, surveillance, and phagocytosis in microglia, and drugs affecting the activity of these genes may be useful in studies of amyloid clearance. We observed extensive dysregulation of gene expression in microglia and other cell types upon loss of *Inpp5d*, but we were surprised by how many of these genes were implicated in diseases other than AD (Figure 3B). Thus, *Inpp5d* expression may affect the ability of microglia to clear protein aggregates in other neurodegenerative diseases.

hdWGCNA is an unsupervised method of identifying groups of genes associated with cell states. We identified a module of genes expressed in microglia centered around *Inpp5d* in female mice (Figure 4E). This module's eigenvalue was dramatically decreased in homozygous knockout mice, confirming that this module is composed of genes whose expression correlates tightly with *Inpp5d*. The genes identified by this analysis may represent attractive candidates for further study of microglial function, particularly in the context of disease. A similar module was present in male mice, but *Inpp5d* was less central, and the loss of *Inpp5d* did not notably decrease the module eigenvalue (Figure S5). These contrasting findings in hdWGCNA between female and male mice, coupled with the elevated number of differentially expressed genes in female *Inpp5d* null mice, suggest that *Inpp5d* may exert greater control over microglial gene regulation and function in female mice than in male mice. Similar observations were made in a study of postmortem human brain tissue, where dysregulation of microglial gene signatures was shown to be more common in female patients than in male patients.^40^

We were also interested in investigating the differences in gene expression in cell types that do not express *Inpp5d*. We used CellChat to infer differences in signaling between microglia and other cell types, which could help explain these findings. This analysis revealed two interesting candidates (PTN and PSAP; Figure 5). Elevated PTN is detected in the substantia nigra of patients with Parkinson's disease and in the cerebrospinal fluid (CSF) of patients with AD.^41^, ^42^ Accumulating evidence points to astrocyte‐derived PTN as an anti‐inflammatory, neuroprotective factor secreted by reactive astrocytes.^43^, ^44^ Downregulation of this signaling pathway through the loss of *Inpp5d* could, therefore, enhance neuroinflammation. Whether neuroinflammation is beneficial or harmful across the course of neurodegeneration remains an important question. Therefore, it will be important to determine whether a decrease in PTN signaling will ameliorate or exacerbate pathology. PSAP is secreted by microglia and astrocytes in humans and predominantly by microglia in mice.^45^, ^46^ In astrocytes, it promotes motility and neuroprotective function, and is upregulated in the brains of patients with AD.^47^, ^48^ These findings indicate that upregulation of PSAP expression in astrocytes and microglia may promote astrocyte reactivity and be neuroprotective. Based on our important identification of PTN and PSAP as possible downstream targets of *Inpp5d*, further study is warranted to determine their roles in astrocytes and AD pathogenesis.

Previous studies of *Inpp5d* in AD have employed APP transgenic or knock‐in models to determine the effect of *Inpp5d* depletion on amyloid pathology.^18^, ^19^, ^20^, ^49^ The methodology used in each study varies: Castranio *et al*. used a *Cx3cr1*‐driven *Cre* conditional knockdown model where tamoxifen was injected at 3 months of age in PSAPP mice. They observed increased amyloid plaque pathology at 6 to 6.5 months of age using immunohistochemistry despite no change in Aβ levels measured by ELISA.^19^ Both sexes were pooled for pathological analysis, but only female mice were used for a spatial transcriptomics experiment. Lin *et al*. studied the effect of *Inpp5d* haploinsufficiency using 5XFAD mice.^18^ In contrast to the study by Castranio *et al.*, the deletion of one copy of *Inpp5d* reduced plaque pathology as measured by immunohistochemical approaches. Both sexes were pooled for amyloid analysis in this study as well, albeit each sex was clearly indicated. A third study by Samuels *et al*. used a conditional knockdown approach in 5XFAD mice.^49^ Tamoxifen was delivered through chow for 2 weeks at 3 weeks of age and tissue was collected at 5 months. Amyloid levels were not changed but microglial number and engagement with plaques increased. In this study, only female mice were used for all readouts. A fourth publication by Iguchi *et al*. using the *APP* NL‐G‐F model studied the effects of *Inpp5d* haploinsufficiency in a TREM‐2 loss‐of‐function model.^20^ At 9 months of age, *Inpp5d* depletion increased microgliosis and plaque engagement in male mice.

Due to the differences across these four studies, it is difficult to draw firm conclusions on the role of *Inpp5d* in Alzheimer's pathology or compare these works with our own. Castriano and Lin used regional spatial transcriptomics. These technologies measure gene expression in regions of a slide‐mounted sample, rather than single cells. Comparisons of regions, cells, and DEGs between technologies should be made cautiously. Samuels *et al*. performed a single‐nucleus 3′‐RNA‐sequencing experiment with *n* = 3 biological replicates pooled into *n* = 1 technical replicate per genotype. Our single‐cell protocol enriches for microglia, whereas single‐nucleus suspensions contain many neuronal nuclei with low numbers of microglia. Iguchi *et al*. provides evidence that *Inpp5d* loss increases glial engagement with plaques, as also observed by both Lin *et al*. and Samuels *et al*. Considering the above, we suggest that future studies of *Inpp5d* include both sexes. We also suggest that *Inpp5d*‐deficient wild‐type littermates are included, to better delineate which effects are specifically relevant to Alzheimer's pathology.

In conclusion, we determined the consequences of a total loss of *Inpp5d* at single‐cell resolution in both male and female mice. The dysregulated genes, otherwise masked by haplosufficiency of *Inpp5d*, will provide new leads for further investigating their roles in microglial function and neurodegenerative diseases.

## CONFLICT OF INTEREST STATEMENT

The authors declare no conflicts of interest. Author disclosures are available in the supporting information.

## CONSENT STATEMENT

No human subjects participated in this study, consent was not necessary.

## Supporting information

Supporting information

Supporting information

Supporting information

Supporting information

Supporting information

Supporting information

Supporting information

Supporting information

Supporting information

Supporting information

Supporting information

Supporting information

Supporting information

Supporting information

Supporting information

Supporting information

Supporting information

Supporting information

Supporting information

Supporting information

Supporting information
