# Supplementary material for: Loss of Inpp5d has disease‐relevant and sex‐specific effects on glial transcriptomes
Source: Alzheimers Dement. 2024 Jun 26;20(8):5311–23. doi: 10.1002/alz.13901 (PMC11350029; doi:10.1002/alz.13901)
Supplement: Supplementary file 1 — Supporting information [file ALZ-20-5311-s004.pdf]

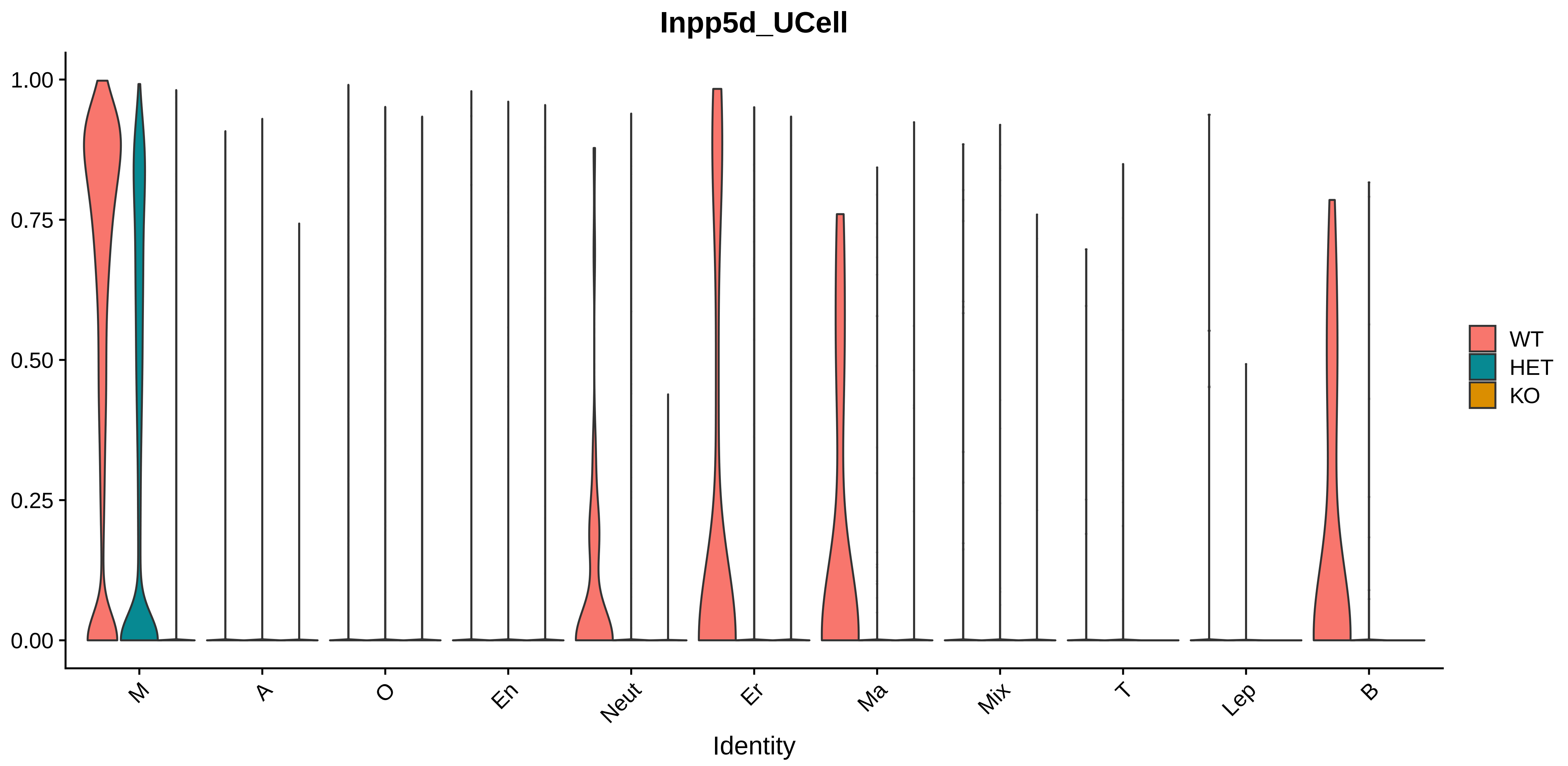

Inpp5d_UCell
1.00
0.75
0.50
0.25
0.00
M
A
O
En
Neut
Er
Ma
Mix
T
Lep
B
Identity
WT
HET
KO

SUPPLEMENTARY FIGURE 1: *Inpp5d* is only expressed in myeloid cells and erythrocytes in the mouse brain. UCell Module scores of *Inpp5d* expression across all cell types identified in Figure 1. *Inpp5d* is not expressed in astrocytes, oligodendrocytes, endothelial or leptomeningeal cells. Heterozygote (HET) and homozygote knockouts (KO) express decreasing levels of *Inpp5d* (to complete depletion in KO mice) compared to wildtype (WT) mice.
