# Supplementary material for: Loss of Inpp5d has disease‐relevant and sex‐specific effects on glial transcriptomes
Source: Alzheimers Dement. 2024 Jun 26;20(8):5311–23. doi: 10.1002/alz.13901 (PMC11350029; doi:10.1002/alz.13901)
Supplement: Supplementary file 2 — Supporting information [file ALZ-20-5311-s018.pdf]

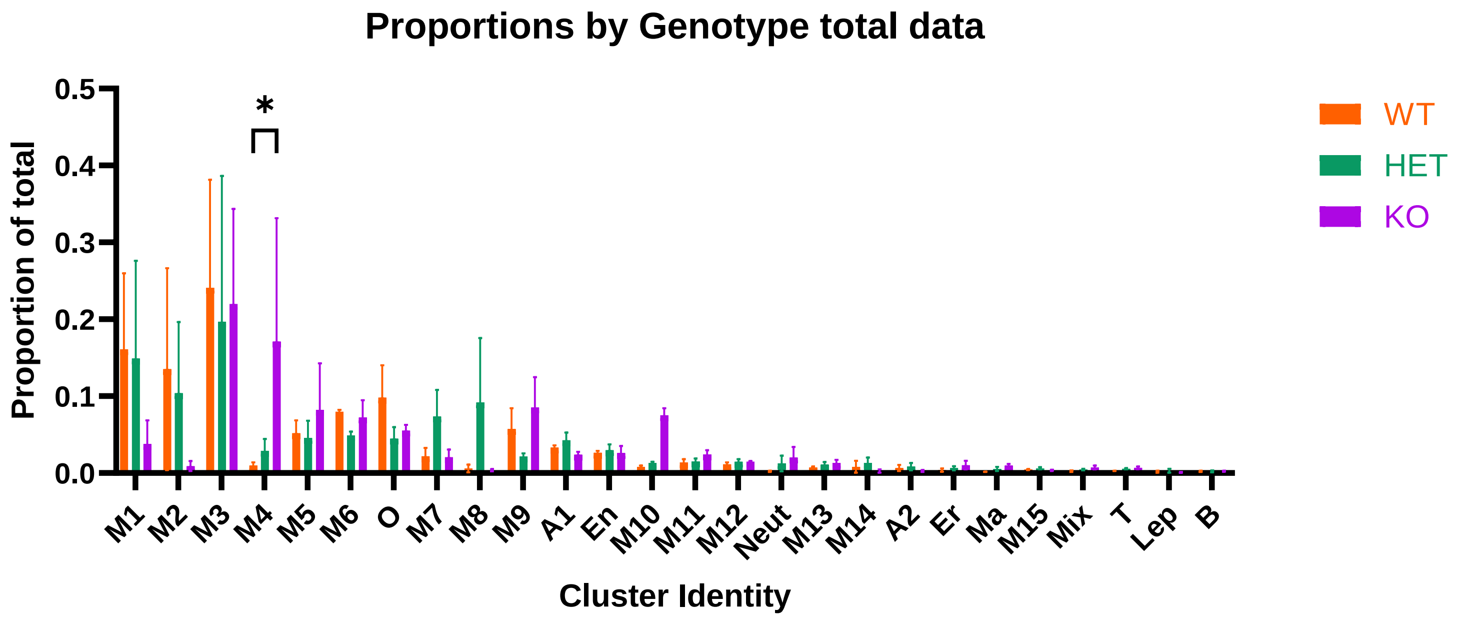

Proportions by Genotype total data
Proportion of total
0.5
0.4
0.3
0.2
0.1
0.0
*
WT
HET
KO
M1
M2
M3
M4
M5
M6
O
M7
M8
M9
A1
En
M10
M11
M12
Neut
M13
M14
A2
Er
Ma
M15
Mix
T
Lep
B
Cluster Identity

SUPPLEMENTARY FIGURE 2: Proportions of cells in each cluster based on *Inpp5d* genotype. ANOVA using Šídák's multiple comparisons test was used to determine nominally significant changes (* = P<0.05 , ** = P<0.01, error bars show SEM).
