# Supplementary material for: Loss of Inpp5d has disease‐relevant and sex‐specific effects on glial transcriptomes
Source: Alzheimers Dement. 2024 Jun 26;20(8):5311–23. doi: 10.1002/alz.13901 (PMC11350029; doi:10.1002/alz.13901)
Supplement: Supplementary file 3 — Supporting information [file ALZ-20-5311-s013.pdf]

Female Microglia Cluster Markers

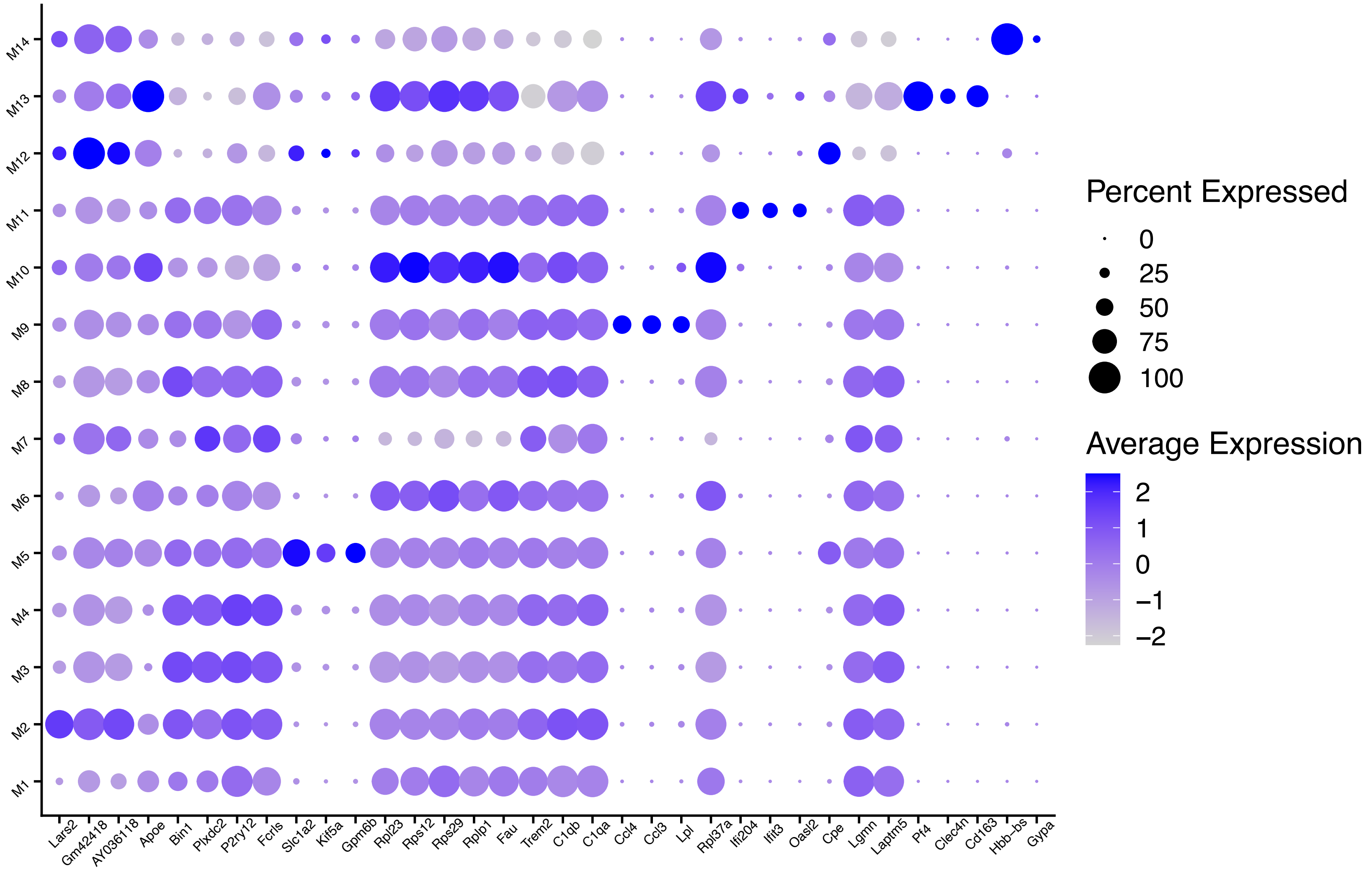

Female Other Cluster Markers

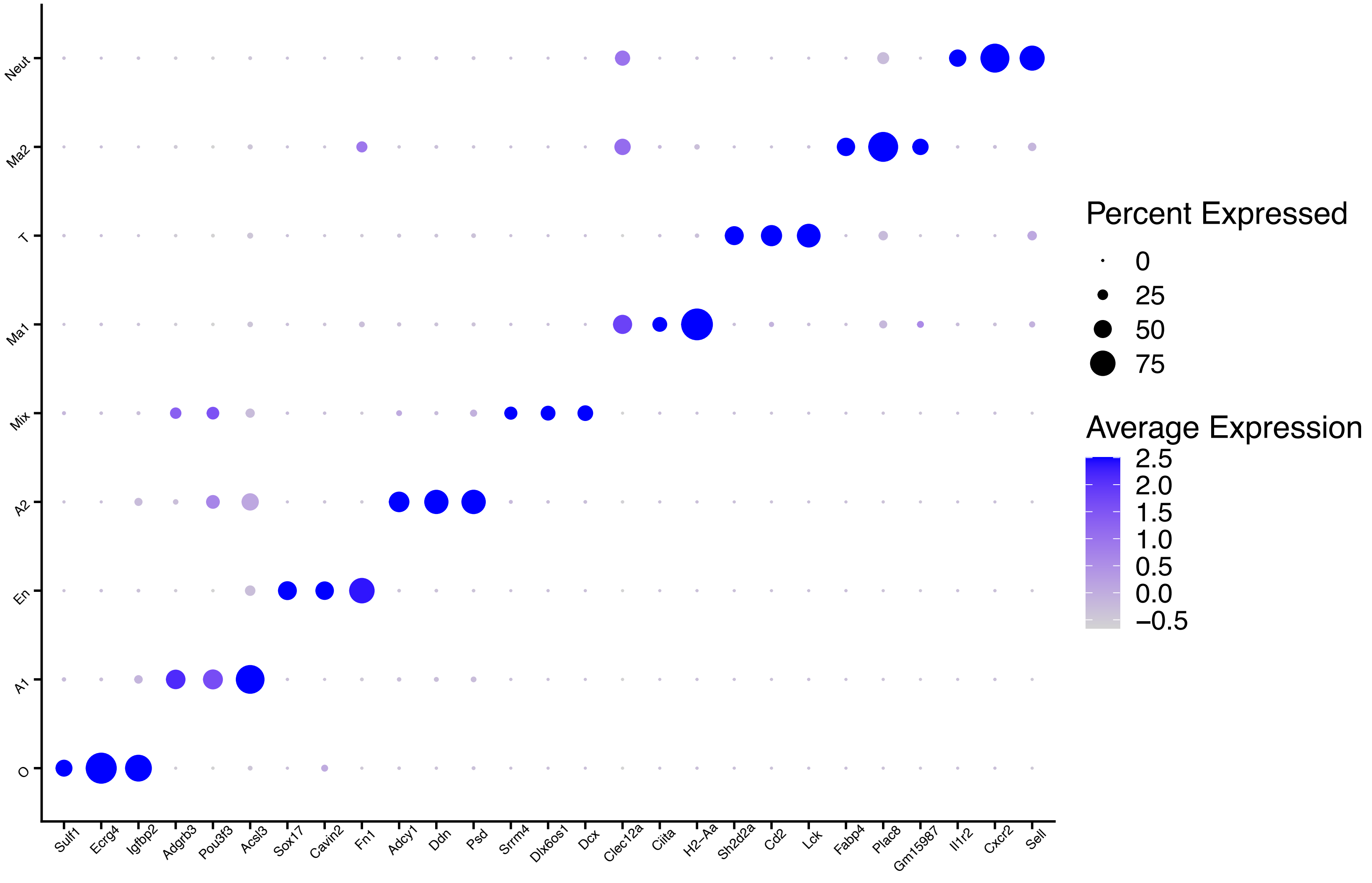

SUPPLEMENTARY FIGURE 3: FEMALE CLUSTER GENES. The y-axes show clusters of microglia (M1-14), and non-microglial cells (Oligodendrocytes, Astrocytes, Endothelial cells, Mixed cells, Macrophages, T-Cells and Neutrophils) and the x-axes show significantly associated marker genes. The size of the dots indicates the percentage of cells in each cluster expressing a particular gene, and the color is shaded based on average expression of that gene compared to the average scaled expression of all genes per cell.
