# Supplementary material for: Loss of Inpp5d has disease‐relevant and sex‐specific effects on glial transcriptomes
Source: Alzheimers Dement. 2024 Jun 26;20(8):5311–23. doi: 10.1002/alz.13901 (PMC11350029; doi:10.1002/alz.13901)
Supplement: Supplementary file 4 — Supporting information [file ALZ-20-5311-s007.pdf]

Male Microglia Cluster Markers

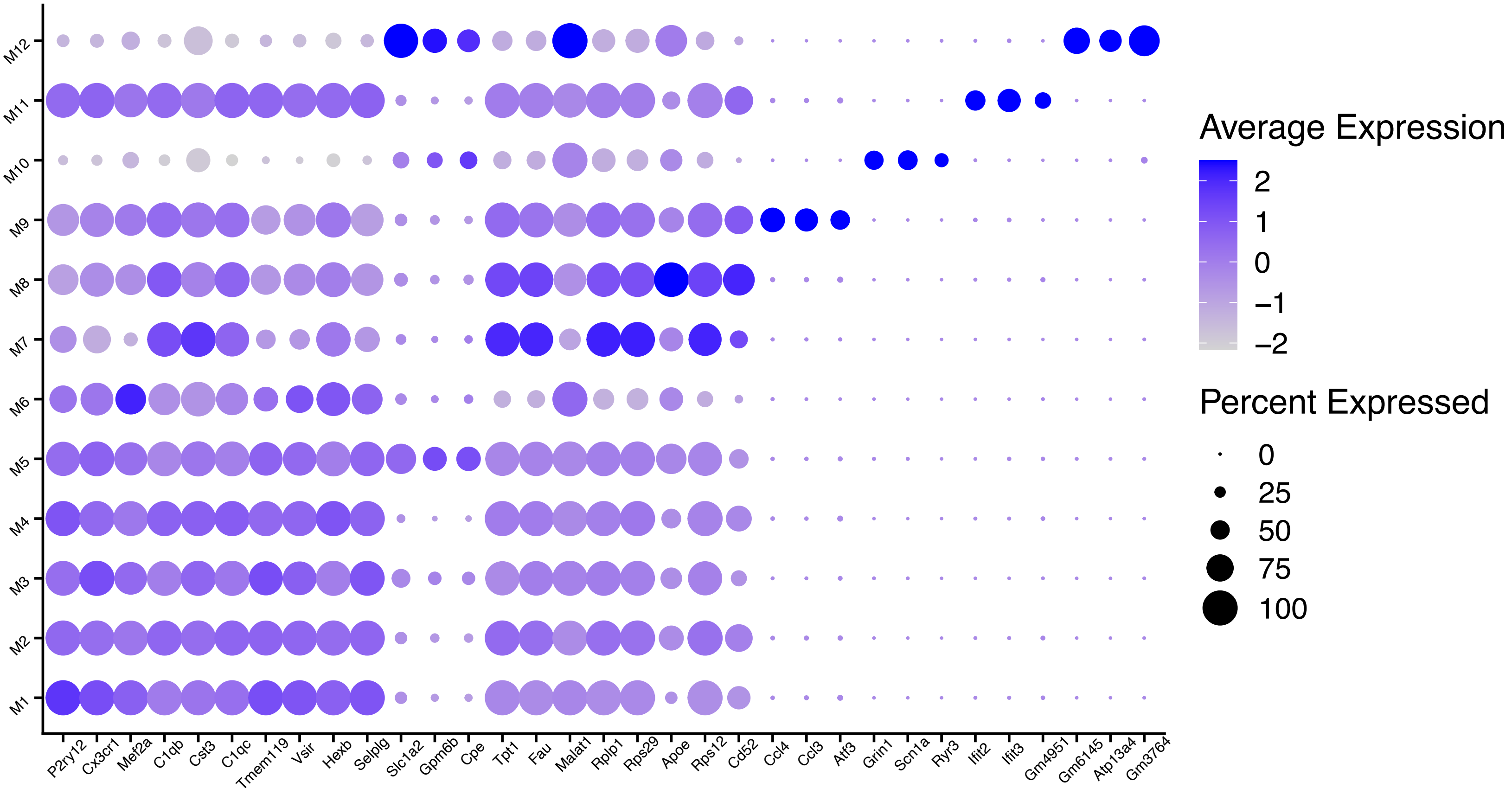

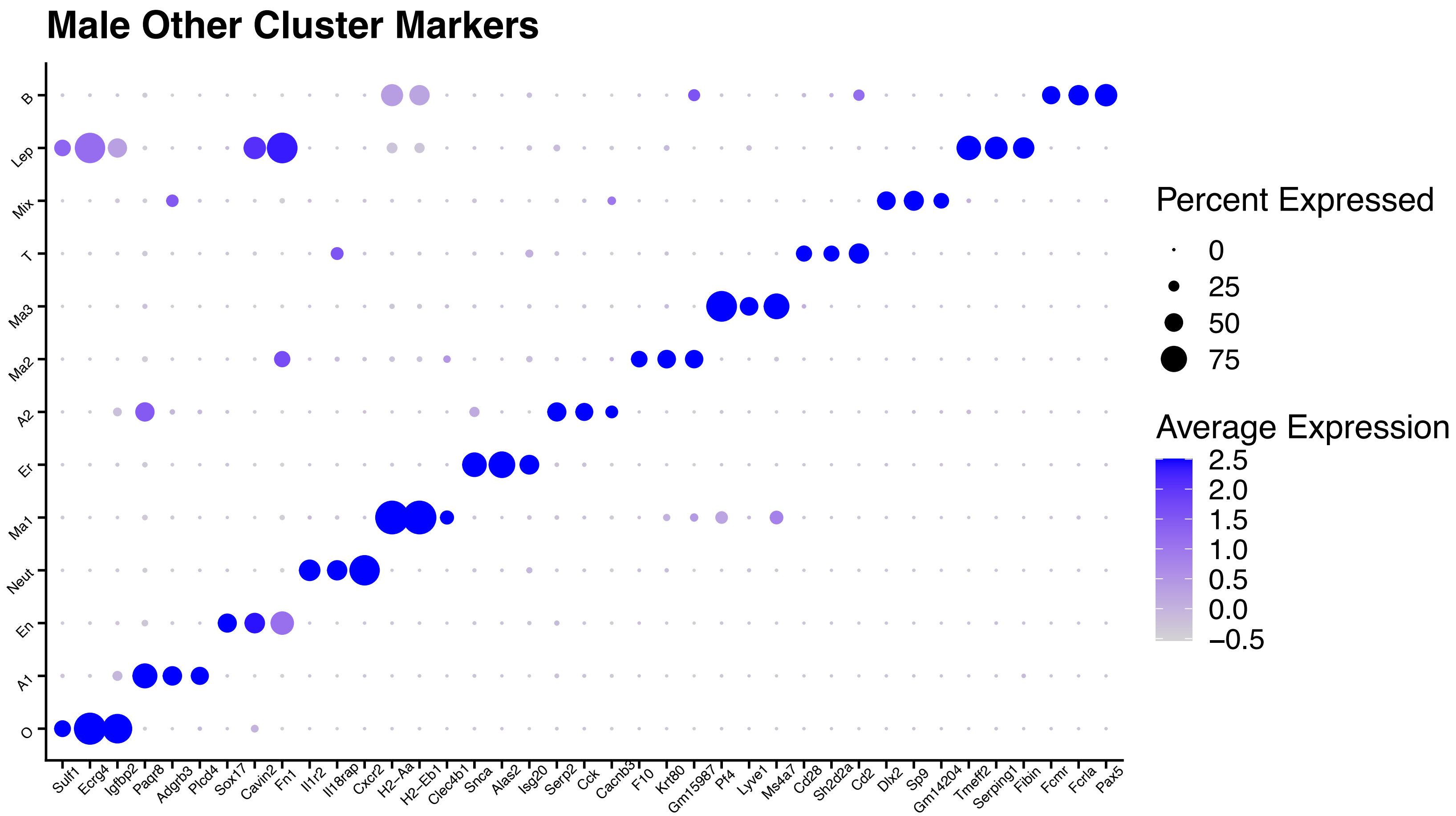
Male Other Cluster Markers
B
Lep
Mix
T
Ma3
Ma2
A2
Er
Ma1
Neut
En
A1
O
Sulf1
Ecrg4
Igfbp2
Paqr8
Adgrb3
Plcd4
Sox17
Cavin2
Fn1
Il1r2
Il18rap
Cxcr2
H2−Aa
H2−Eb1
Clec4b1
Snca
Alas2
Isg20
Serp2
Cck
Cacnb3
F10
Krt80
Gm15987
Pf4
Lyve1
Ms4a7
Cd28
Sh2d2a
Cd2
Dlx2
Sp9
Gm14204
Tmeff2
Serping1
Fibin
Fcmr
Fcrla
Pax5
Percent Expressed
0
25
50
75
Average Expression
2.5
2.0
1.5
1.0
0.5
0.0
−0.5

SUPPLEMENTARY FIGURE 4: MALE CLUSTER GENES. The y-axes show clusters of microglia (M1-12), and non-microglial cells (Oligodendrocytes, Astrocytes, Endothelial cells, Neutrophils, Macrophages, Erythrocytes, T-cells, Mixed cells, Leptomeningeal cells and B-cells and) and the x-axes show significantly associated marker genes. The size of the dots indicates the percentage of cells in each cluster expressing a particular gene, and the color is shaded based on average expression of that gene compared to the average scaled expression of all genes per cell.
