# Supplementary material for: Loss of Inpp5d has disease‐relevant and sex‐specific effects on glial transcriptomes
Source: Alzheimers Dement. 2024 Jun 26;20(8):5311–23. doi: 10.1002/alz.13901 (PMC11350029; doi:10.1002/alz.13901)
Supplement: Supplementary file 5 — Supporting information [file ALZ-20-5311-s015.pdf]

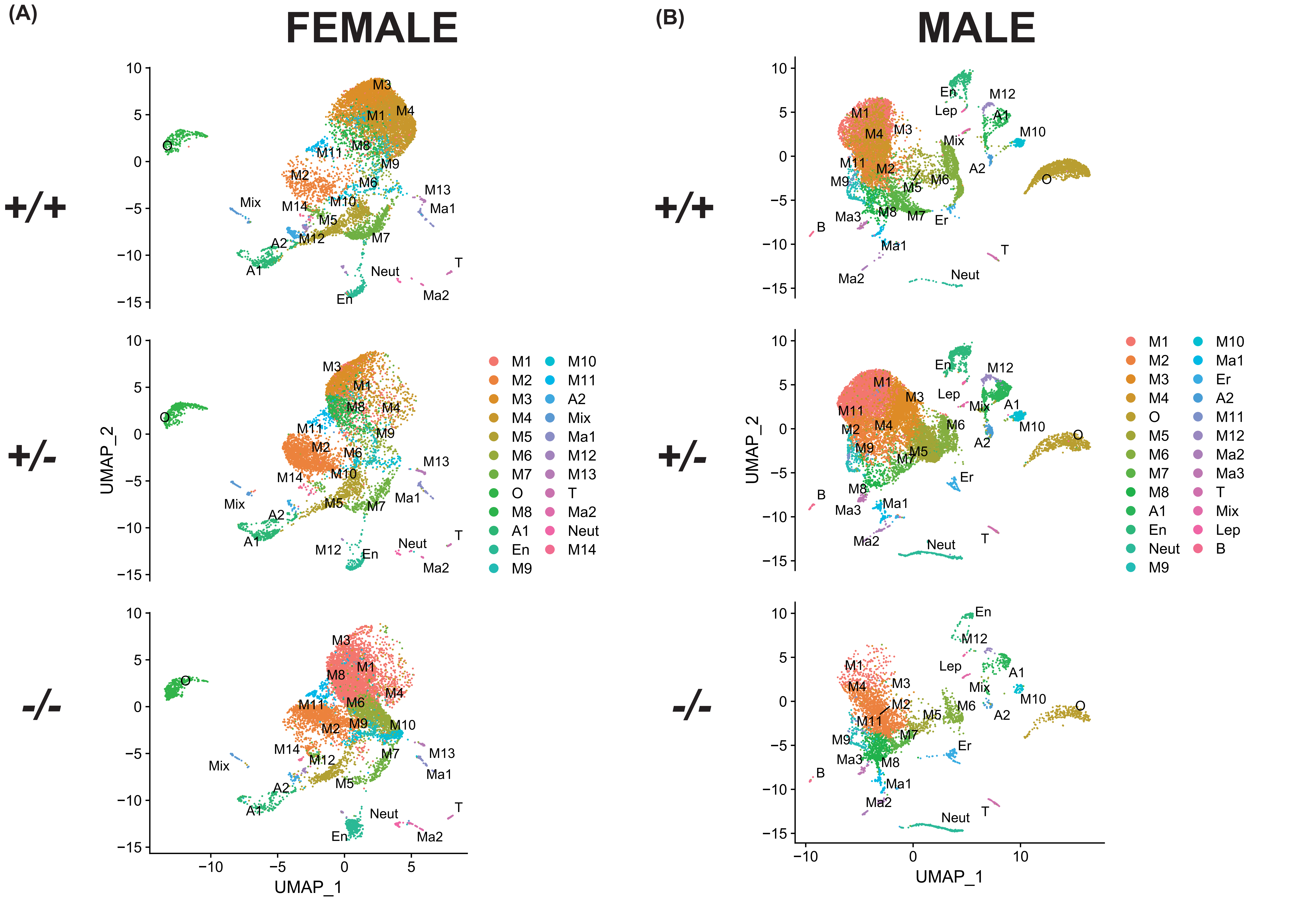
(A)
FEMALE
(B)
MALE
+/+
+/-
-/-
UMAP_1
UMAP_2
M1
M2
M3
M4
M5
M6
M7
O
M8
A1
En
M9
M10
M11
A2
Mix
Ma1
M12
M13
T
Ma2
Neut
M14
Er
Ma3
Lep
B

SUPPLEMENTARY FIGURE 5: UMAPS PER GROUP. (A) UMAP showing changes in cell distributions between clusters in female *Inpp5d* wildtype (+/+), heterozygous (+/-) and homozygous knockout (-/-) mice. (B) UMAP showing changes in cell distributions between clusters in male *Inpp5d* wildtype (+/+), heterozygous (+/-) and homozygous knockout (-/-) mice. (A = astrocytes, B = B-cells, En = endothelial cells, Er = erythrocytes, Lep = leptomeningeal cells, M = microglia, Ma = macrophage, Mix = mixed cells, Neut = neutrophils, O = oligodencrocytes, T =T-cells)
