# Supplementary material for: Loss of Inpp5d has disease‐relevant and sex‐specific effects on glial transcriptomes
Source: Alzheimers Dement. 2024 Jun 26;20(8):5311–23. doi: 10.1002/alz.13901 (PMC11350029; doi:10.1002/alz.13901)
Supplement: Supplementary file 6 — Supporting information [file ALZ-20-5311-s020.pdf]

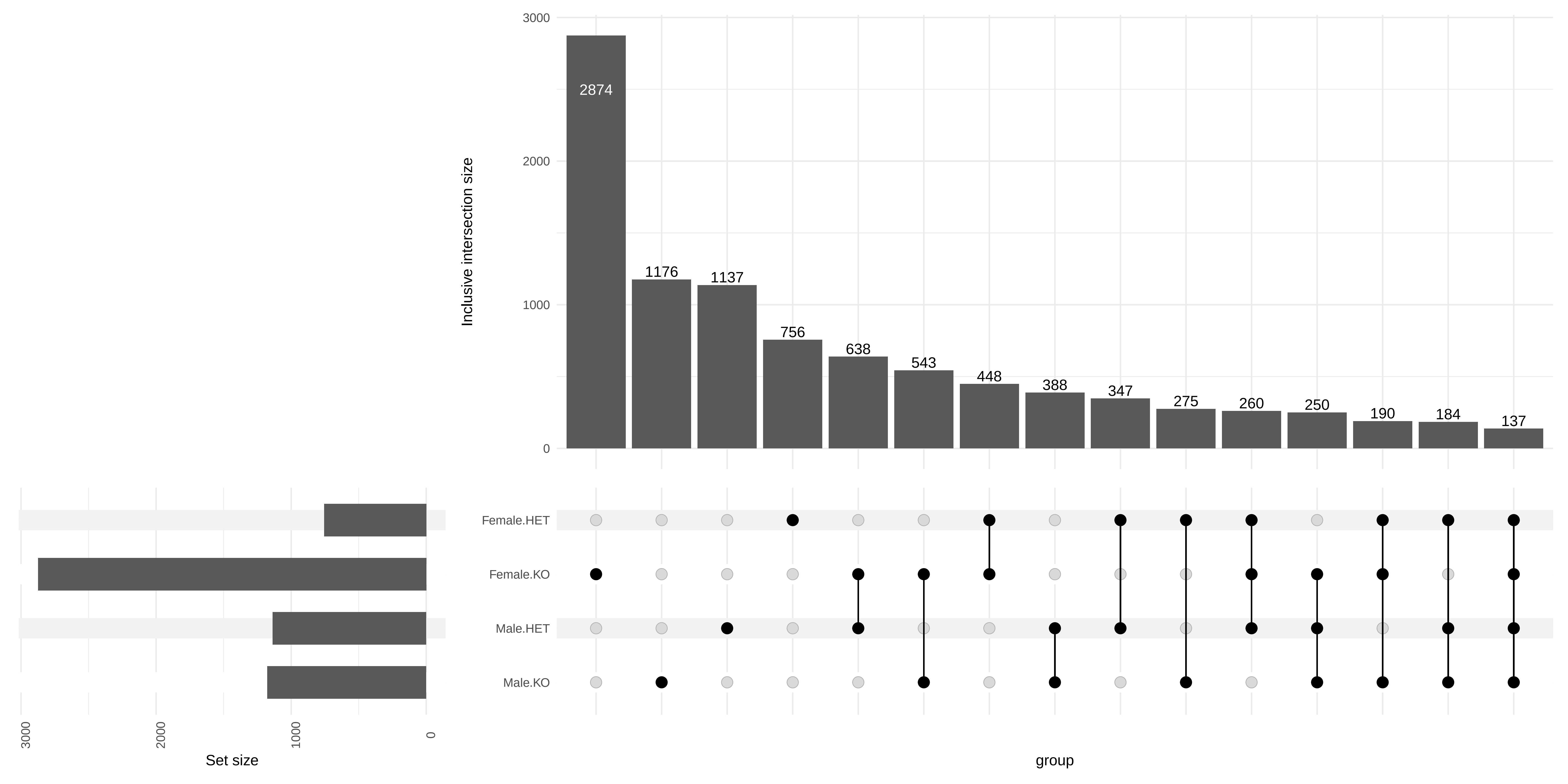

Inclusive intersection size
3000
2000
1000
0
2874
1176
1137
756
638
543
448
388
347
275
260
250
190
184
137
Female.HET
Female.KO
Male.HET
Male.KO
3000
2000
1000
0
Set size
group

SUPPLEMENTARY FIGURE 6: Upset Plot showing the number of unique differentially expressed genes (Set size) across all clusters within each group compared to wildtype and the size of the overlap between all comparisons. Differential expression was determined by Wilcoxon Rank Sum test with Bonferroni adjustment for multiple comparisons (HET = Heterozygous knockout, KO = Homozygous knockout).
