# Supplementary material for: Loss of Inpp5d has disease‐relevant and sex‐specific effects on glial transcriptomes
Source: Alzheimers Dement. 2024 Jun 26;20(8):5311–23. doi: 10.1002/alz.13901 (PMC11350029; doi:10.1002/alz.13901)
Supplement: Supplementary file 7 — Supporting information [file ALZ-20-5311-s005.pdf]

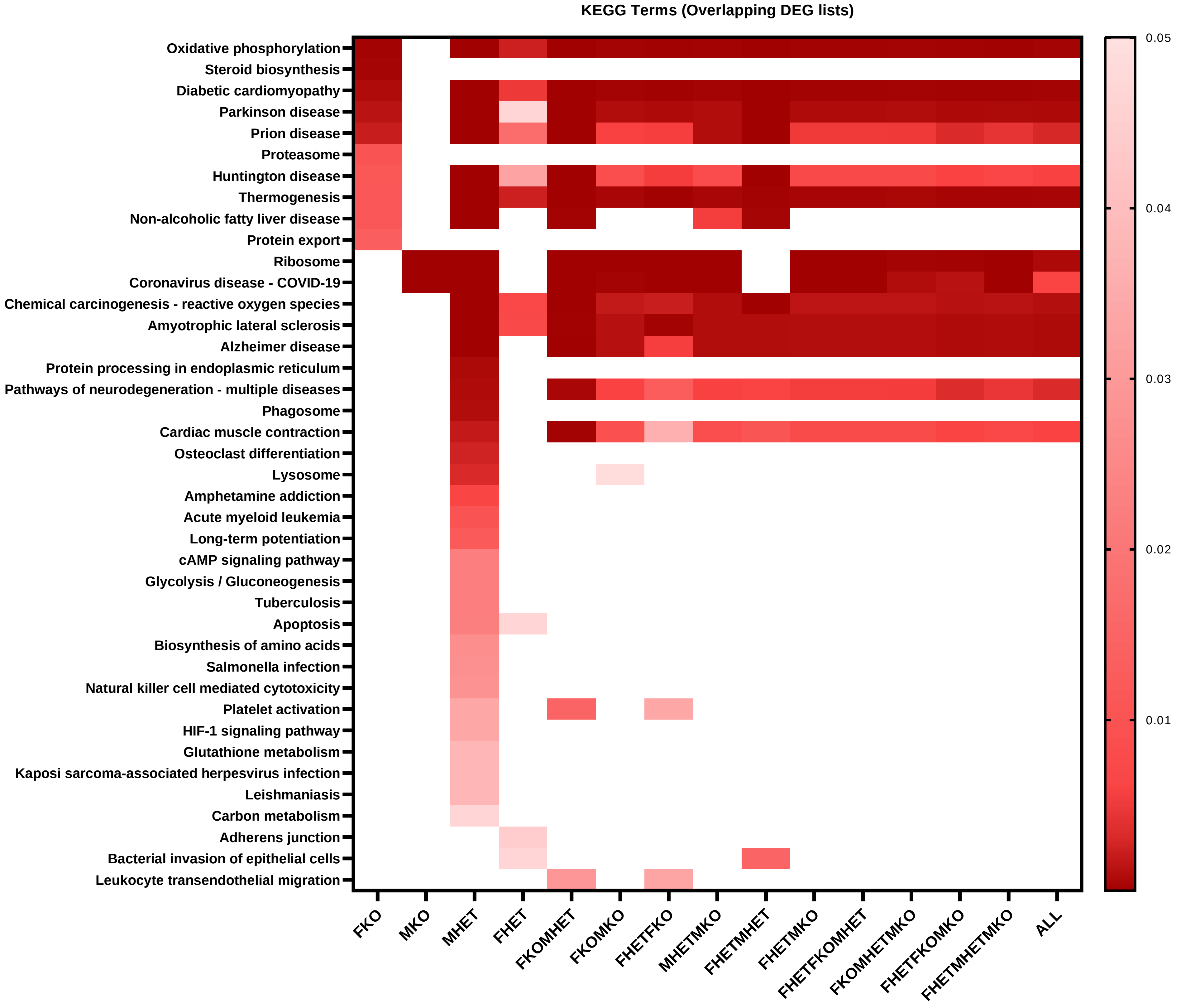

KEGG Terms (Overlapping DEG lists)
Oxidative phosphorylation
Steroid biosynthesis
Diabetic cardiomyopathy
Parkinson disease
Prion disease
Proteasome
Huntington disease
Thermogenesis
Non-alcoholic fatty liver disease
Protein export
Ribosome
Coronavirus disease - COVID-19
Chemical carcinogenesis - reactive oxygen species
Amyotrophic lateral sclerosis
Alzheimer disease
Protein processing in endoplasmic reticulum
Pathways of neurodegeneration - multiple diseases
Phagosome
Cardiac muscle contraction
Osteoclast differentiation
Lysosome
Amphetamine addiction
Acute myeloid leukemia
Long-term potentiation
cAMP signaling pathway
Glycolysis / Gluconeogenesis
Tuberculosis
Apoptosis
Biosynthesis of amino acids
Salmonella infection
Natural killer cell mediated cytotoxicity
Platelet activation
HIF-1 signaling pathway
Glutathione metabolism
Kaposi sarcoma-associated herpesvirus infection
Leishmaniasis
Carbon metabolism
Adherens junction
Bacterial invasion of epithelial cells
Leukocyte transendothelial migration
FKO
MKO
MHET
FHET
FKOMHET
FKOMKO
FHETFKO
MHETMKO
FHETMHET
FHETMKO
FHETFKOMHET
FKOMHETMKO
FHETFKOMKO
FHETMHETMKO
ALL
0.05
0.04
0.03
0.02
0.01

SUPPLEMENTARY FIGURE 7: Heatmap of Gene Ontology enrichment analysis of all lists of DEGs shown in Extended Figure 3 with KEGG terms (P-value adjustment performed using the Benjamini-Hochberg method, all significantly enriched terms are shown per comparison – none were excluded).
