# Supplementary material for: Loss of Inpp5d has disease‐relevant and sex‐specific effects on glial transcriptomes
Source: Alzheimers Dement. 2024 Jun 26;20(8):5311–23. doi: 10.1002/alz.13901 (PMC11350029; doi:10.1002/alz.13901)
Supplement: Supplementary file 8 — Supporting information [file ALZ-20-5311-s011.pdf]

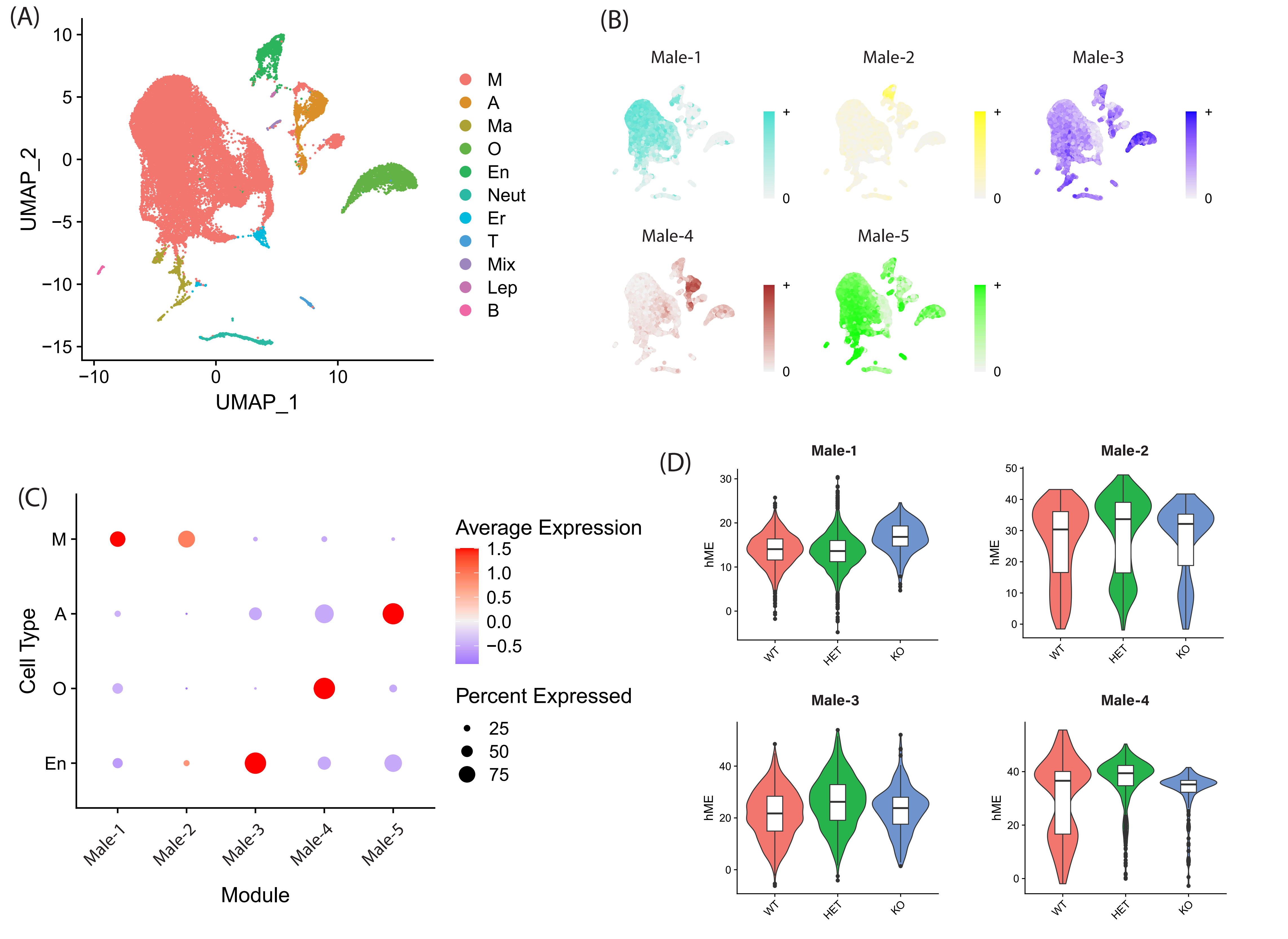

(A)
UMAP_2
UMAP_1
M
A
Ma
O
En
Neut
Er
T
Mix
Lep
B
(B)
Male-1
Male-2
Male-3
Male-4
Male-5
(C)
Cell Type
Module
Average Expression
Percent Expressed
(D)
hME
WT
HET
KO

**SUPPLEMENTARY FIGURE 8: High-dimensional WGCNA reveals cell-type specific co-expression gene modules in male mice, including a microglial module with *Inpp5d* at its center.** (A) UMAP showing 11 clusters of merged cell-types. The four largest clusters - microglia (M), astrocytes (A), oligodendrocytes (O) and endothelial cells (En) - were used in this analysis. (B) Feature plot of expression of Hub genes (top 10% of genes as ranked using Maximal Clique Centrality) per module (Male 1-5). (C) Dotplot showing scaled expression of each module across cell types. Average scaled expression level scales with blue (low) to red (high) shading. Size of dots represents the percent of cells in each cell type expressing the module. (D) Eigenvalues of the first four modules across genotypes.
