# Supplementary material for: Loss of Inpp5d has disease‐relevant and sex‐specific effects on glial transcriptomes
Source: Alzheimers Dement. 2024 Jun 26;20(8):5311–23. doi: 10.1002/alz.13901 (PMC11350029; doi:10.1002/alz.13901)
Supplement: Supplementary file 9 — Supporting information [file ALZ-20-5311-s003.pdf]

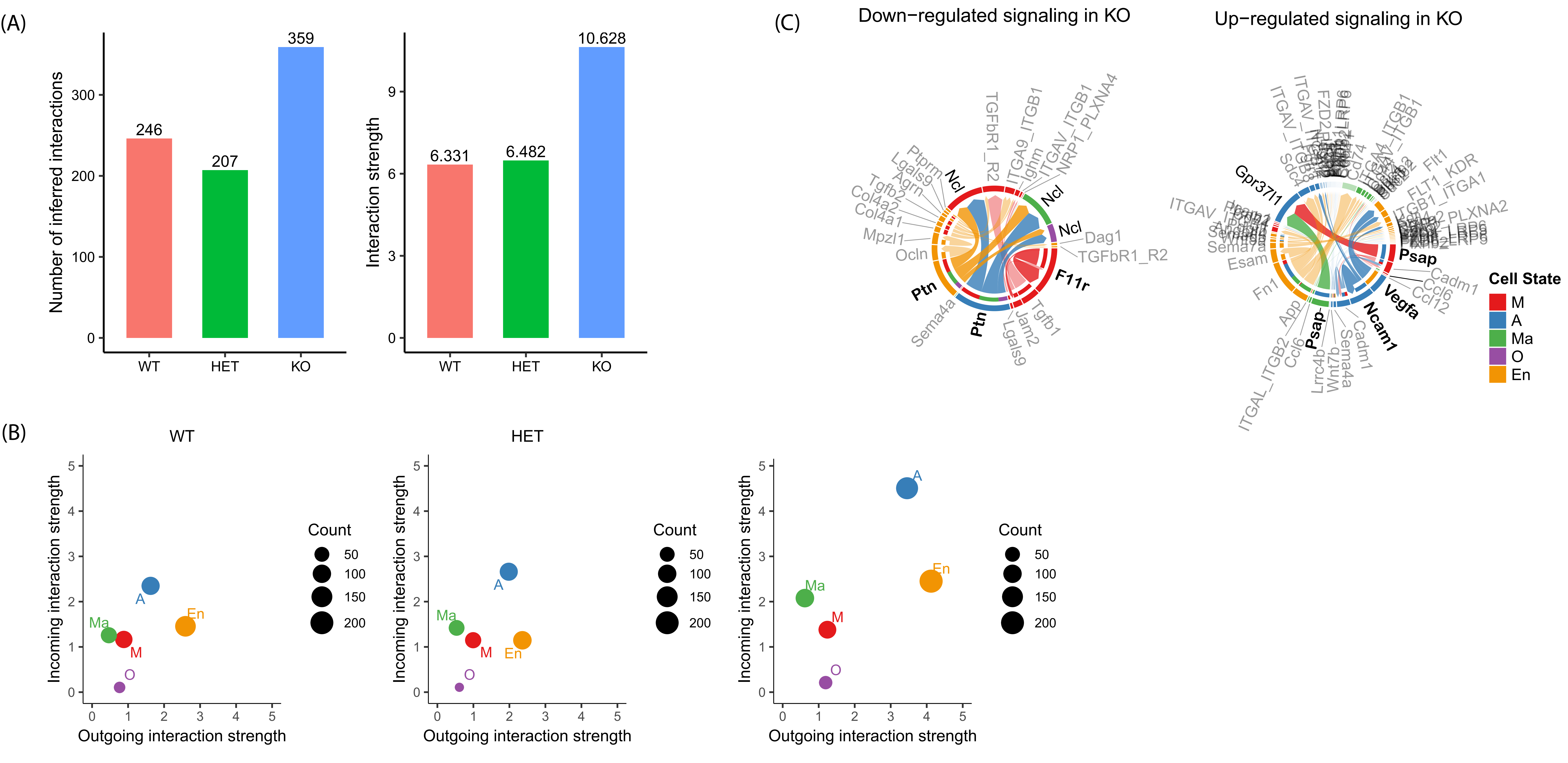

(A)
Number of inferred interactions
246
207
359
WT
HET
KO
Interaction strength
6.331
6.482
10.628
(C)
Down−regulated signaling in KO
Up−regulated signaling in KO
Ncl
Ptn
F11r
Psap
Vegfa
Ncam1
Gpr37l1
Cell State
M
A
Ma
O
En
(B)
WT
HET
Incoming interaction strength
Outgoing interaction strength
Count
50
100
150
200

SUPPLEMENTARY FIGURE 9: Total loss of *Inpp5d* increases global signalling between cell types in male mice. (A The number and strength of inferred interactions increases on homozygous loss of *Inpp5d* in male mice. (B) Numbers and strengths of incoming and/or outgoing interactions increase in all cell types on total loss of *Inpp5d* in male mice. (C) Chord plots showing pathways with decreased and increased signalling in *Inpp5d*$^{-/-}$ mice compared to wildtype. Plot circumference shows outgoing signalling from cell type (outer band) to recipient cell type (inner band). Width of outer bands represents numbers of cells per pathway. All pathways shown are significantly dysregulated, some bands are emphasised for the purpose of visualisation.
